# Evaluating Convolutional Neural Networks for Cage-Free Floor Egg Detection

**DOI:** 10.3390/s20020332

**Published:** 2020-01-07

**Authors:** Guoming Li, Yan Xu, Yang Zhao, Qian Du, Yanbo Huang

**Affiliations:** 1 Department of Agricultural and Biological Engineering, Mississippi State University, Starkville, MS 39762, USA; gl565@msstate.edu; 2 Department of Electrical and Computer Engineering, Mississippi State University, Starkville, MS 39762, USA; yanxumsu@gmail.com (Y.X.); du@ece.msstate.edu (Q.D.); 3 Agricultural Research Service, Crop Production Systems Research Unit, United States Department of Agriculture, Stoneville, MS 38776, USA; yanbo.huang@usda.gov

**Keywords:** floor egg, convolutional neural network, tensorflow, cage-free, evaluation

## Abstract

The manual collection of eggs laid on the floor (or ‘floor eggs’) in cage-free (CF) laying hen housing is strenuous and time-consuming. Using robots for automatic floor egg collection offers a novel solution to reduce labor yet relies on robust egg detection systems. This study sought to develop vision-based floor-egg detectors using three Convolutional Neural Networks (CNNs), i.e., single shot detector (SSD), faster region-based CNN (faster R-CNN), and region-based fully convolutional network (R-FCN), and evaluate their performance on floor egg detection under simulated CF environments. The results show that the SSD detector had the highest precision (99.9 ± 0.1%) and fastest processing speed (125.1 ± 2.7 ms·image^−1^) but the lowest recall (72.1 ± 7.2%) and accuracy (72.0 ± 7.2%) among the three floor-egg detectors. The R-FCN detector had the slowest processing speed (243.2 ± 1.0 ms·image^−1^) and the lowest precision (93.3 ± 2.4%). The faster R-CNN detector had the best performance in floor egg detection with the highest recall (98.4 ± 0.4%) and accuracy (98.1 ± 0.3%), and a medium prevision (99.7 ± 0.2%) and image processing speed (201.5 ± 2.3 ms·image^−1^); thus, the faster R-CNN detector was selected as the optimal model. The faster R-CNN detector performed almost perfectly for floor egg detection under a wide range of simulated CF environments and system settings, except for brown egg detection at 1 lux light intensity. When tested under random settings, the faster R-CNN detector had 91.9–94.7% precision, 99.8–100.0% recall, and 91.9–94.5% accuracy for floor egg detection. It is concluded that a properly-trained CNN floor-egg detector may accurately detect floor eggs under CF housing environments and has the potential to serve as a crucial vision-based component for robotic floor egg collection systems.

## 1. Introduction

The US egg industry is transitioning to alternative hen housing systems due to subpar bird welfare conditions in conventional cage housing systems. Cage-free (CF) housing systems are among the alternative systems that provide hens with larger living spaces and welfare enrichments, such as perches, nestboxes, and litter floor [1]. While hens have accesses to a litter floor in CF systems, they may lay eggs on the floor [2,3], namely floor eggs. Floor eggs represent approximately 0.2–2% of daily egg production, even with proper animal training and management [4]. In some extreme cases (e.g., lack of training for nesting, accidental nestbox access restriction, etc.), floor eggs could exceed 5% of total egg production [3,4], translating to over 2500 daily floor eggs in a typical 50,000-hen CF house. Because floor eggs are directly contacted with litter/manure and exposed to hens, they may be contaminated and/or pecked by birds if not collected in a timely manner [5]. Furthermore, floor eggs may induce egg eating, degradation of egg quality, and risk of additional floor egg laying [6]. Currently, floor eggs require manual collection that is time-consuming and laborious. Automatic floor egg collection is among the primary interests for egg producers to sustain production in CF housing. One potential solution is to collect floor eggs using robots.

Robotic systems have been developed and integrated for various agricultural applications [4,7,8,9]. The robotic systems typically consisted of vision-based detectors to detect objects of concern. Some detectors developed based on traditional image analysis may require a large set of parameters and reduce their detection accuracy when used in variable and complex environments [7,9]. The environments and conditions of floor egg detection in CF housing are complicated and may vary in terms of light distribution, egg cleanness, egg color, egg size, etc. [10]. Robust detectors that can handle such complex situations in CF housing are, therefore, needed for the accurate detection of floor eggs. 

Convolutional neural networks (CNNs) have been widely used for object detection [11]. With CNN-based detectors, a series of features is firstly extracted from an input image and fed into the detectors. Then, objects of concern in the image can be identified based on probability of feature matching [11]. Quite a few CNNs exist, which may be categorized by function, architecture, and performance. Among them, single shot detector (SSD), faster region-based CNN (faster R-CNN), and region-based fully convolutional network (R-FCN) have been widely used to detect a variety of objects. Specifically, SSD is a feedforward convolutional network which can directly and quickly detect the presence of objects by live streaming [12]. Faster R-CNN and R-FCN use regional information to detect objects in images/videos at the expense of the processing speed [13,14]. Wang, et al. [15] developed a faster R-CNN detector to detect dairy goats in a surveillance video and obtained 92.5% average precision. Yang, et al. [16] used faster R-CNN to locate and identify individual pigs from a group-housed pen, calculated the feeding area occupation rate to detect pig feeding behaviors, and achieved 99.6% precision and 86.9% recall for pig feeding behavior detection. Nasirahmadi, et al. [17] compared the faster R-CNN, SSD, and R-FCN on pig standing and lying posture detection under commercial farm conditions, and found that the R-FCN can detect pigs standing and lying with higher average precision (more than 92%) and mean average precision (more than 93%). With such high performance on object detection in precision livestock farming, the three CNNs may have the potential to detect floor eggs under various CF housing environments, although the detection performance remains unclear. 

The objectives of this study were to (1) develop vision-based floor-egg detectors based on three CNNs, i.e. SSD, faster R-CNN, and R-FCN; (2) compare the performance of the three CNN floor-egg detectors to detect floor eggs under a range of simulated CF environments and system settings; (3) evaluate performance of the optimal CNN floor-egg detector under different settings; and (4) assess the generalizability of the optimal CNN floor-egg detector under random settings. Processing speed, precision, recall, accuracy, and root mean square error (RMSE) were used as the criteria for evaluating the performance of the detectors. This work will provide a CNN-based floor egg detector for floor egg detection and some useful insights into developing egg-collecting robots when the detector is integrated therein.

## 2. Materials and Methods

### 2.1. System Description

**Figure 1:**
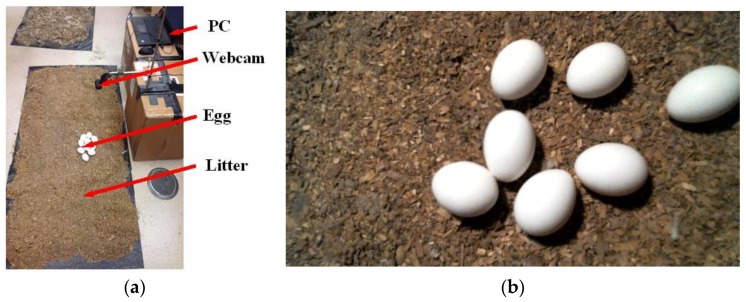
A photo of the imagery system setup (**a**) and a top-view sample image of floor eggs (**b**).

The experiment was conducted in the Instrumentation Lab of the Department of Agricultural and Biological Engineering, Mississippi State University, Mississippi state, USA. Figure 1a shows the imagery system for this experiment. A HD C615-Black webcam (Logitech International S.A., Silicon Valley, CA) was used to acquire images of 1920 × 1080 pixels each (Figure 1b). Litter was obtained from a commercial CF farm. The computer system used for detector training, validation, and testing computing was equipped with 32 GB RAM, Intel(R) Core (TM) i7-8700K processor, and NVIDIA GeForce GTX 1080 GPU card (Dell Inc., Round Rock, TX, USA). The Python programming language (v3.6.8), TensorFlow-GPU (v1.13.1), an open source machine learning library with cuDNN (v7.4), a GPU-accelerated library of primitives for deep neural networks, and a powerful software development platform for building GPU-accelerated applications CUDA (v10.0) were used. Brown and white eggs for the tests were procured from a local grocery store (Walmart, Starkville, MS, USA).

### 2.2. Network Description

In this study, we developed floor-egg detectors using three CNNs, i.e., SSD, faster R-CNN, and R-FCN, due to their common use in object detection [18]. The SSD, faster R-CNN, and R-FCN were, respectively, associated with the Mobilenet V1 [19], Inception V2 [20], and Resnet101 [21] feature extractors, which differ in structure and complexity [11].

**Figure 2:**
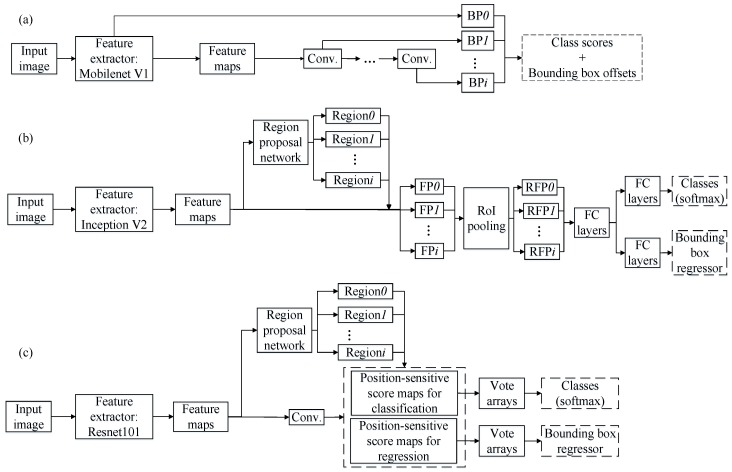
Schematic illustration of (**a**) single shot detector, (**b**) faster region-based convolutional neural network, and (**c**) region-based fully connected network. Conv. is convolutional layer, BP*_i_* is the *i*th box predictor, Region*_i_* is the *i*th region proposal, FP*_i_* is the *i*th feature patch, RFP*_i_* is the *i*th resized feature patch, and FC layers are fully-connected layers.

The SSD produces a set of bounding boxes and scores for the presence of objects by using a feed-forward convolutional network (Figure 2a). Its feature extractor (Mobilenet V1) is used as the base network to extract the main features from original images, and the operation of the extractor consists of multiple steps of depthwise and pointwise convolutions. Then, different types of filters are applied to generate multiscale feature maps. A series of default bounding boxes is assigned to each location on these feature maps, and the box sizes are adjusted to match the sizes of these feature maps. Scores of all classes and offsets are predicted for different ratios of the modified default box. Finally, with nonmaximum suppression, the highest score of a class is maintained, and the offsets are used to adjust the bounding box accordingly.

The faster R-CNN uses a unified neural network for both region proposal and object detection tasks (Figure 2b). Unlike the predecessors (R-CNN and fast R-CNN), the faster R-CNN avoids using selective search to find region proposals, which can speed up region selections and further reduce computation cost. The faster R-CNN detector mainly consists of a regional proposal network (RPN), generating region proposals, and a network using these proposals for object detection. The input image is passed through the feature extractor (Inception V2) containing multiple-size filters, and the resultant features are concatenated together to generate the feature maps. The RPN takes the feature maps as the input, and outputs a set of regions, which are tiled onto the feature maps to crop a series of small feature patches. A region of interest (RoI) pooling layer is used to wrap these patches into fixed sizes. Finally, the resized feature patches are joined with a set of fully connected (FC) layers, and two additional separated FC layers are used to predict object scores and refine locations.

The R-FCN is a region-based, fully-convolutional network (Figure 2c), which can detect objects by using relative spatial information. Its Resnet101 feature extractor applied to extract feature maps contains the 101-layer residual network and is formed by skipping connections among layers that can optimize detection performance in the deep connections of a network. Then, the feature maps are shared by both a fully-convolutional network (FCN) and an RPN. Two types of position-sensitive score maps for classification and regression, which encode the relative spatial information (e.g., top left, top center, top right, etc.), are generated after the feature maps are passed though the FCN. The RPN proposes candidate RoIs, which are applied to the score maps. A pooling layer and an average voting strategy are performed to generate the vote arrays for classifying objects and refining the locations of the bounding boxes.

### 2.3. General workflow of Detector Training, Validation, and Testing

**Figure 3:**
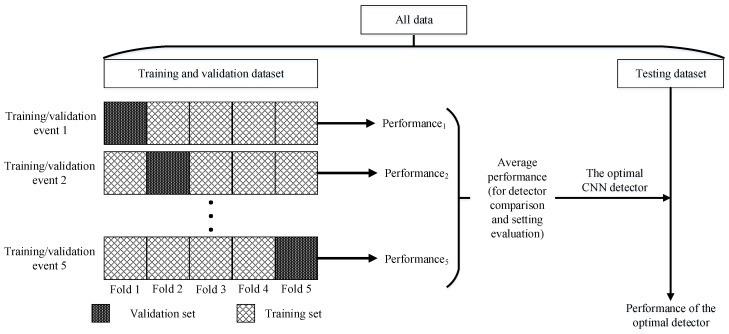
Illustration of the training, validation, and testing process. CNN means convolutional neural network. Performance includes precision, recall, accuracy, RMSE, and processing speed.

A five-fold cross validation strategy was used to evaluate the detectors in this study (Figure 3). The dataset was split into two, with one for training and validation, and the other for testing. The training and validation dataset was then randomly divided into five equal folds. The details of the sample size for each dataset will be presented later. For each training/validation event, the CNN detectors (i.e., SSD, faster RCNN, and R-FCN) were trained using four of the five folds as training data, and the resultant detectors were validated using the rest fold. The performance (e.g., precision, recall, accuracy, RMSE, and processing speed) of the detectors was averaged to determine the optimal CNN detector, which was finally evaluated with the testing dataset to test the generalizability of the optimal detector. The performance of the optimal detector was also calculated accordingly.

### 2.4. Development of CNN Floor-Egg Detectors

#### 2.4.1. Preparation of Development Environment

An open source framework, the Google TensorFlow Object Detection Application Programming Interface, provided the three CNNs of concern [22]. The three CNNs were pretrained using the Common Objects in Context (COCO) dataset and may be readily modified into the desired object detectors through network training. Before developing the floor-egg detectors, the development environment was prepared according to the following steps:

- Install libraries and accessories including Python, Pillow, Lxml, Cython, Matplotlib, Pandas, OpenCV, and TensorFlow-GPU. This step creates the appropriate virtual environment for detector training, validation, and testing.
- Label eggs in images and create .xml (XML) files. A Python-based annotation tool, LabelImg, is used to label eggs in images with rectangular bounding boxes. The labels are saved as XML files in Pascal Visual Object Class format, which contain file name, file path, image size (width, length, and depth), object identification, and pixel coordinates (x_min_, y_min_, x_max_, and y_max_) of the bounding boxes. Each image corresponds to one XML file.
- Create .csv (CSV) and TFRecord files. The CSV files contain image name, image size (width and length, and depth), object identification, and pixel coordinates (x_min_, y_min_, x_max_, and y_max_) of all bounding boxes in each image. The CSV files are then converted into TFRecord files which follow TensorFlow’s binary storage formats.
- Install CNN pretrained object detectors downloaded from TensorFlow detection model zoo [18]. The versions of the detectors were “ssd_mobilenet_v1_coco_2018_01_28” for the SSD detector, “faster_rcnn_inception_v2_coco_2018_01_28” for the faster R-CNN detector, and “rfcn_resnet101_coco_2018_01_28” for the R-FCN detector in this study.

#### 2.4.2. Development of the Floor-Egg Detectors (Network Training)

**Figure 4:**
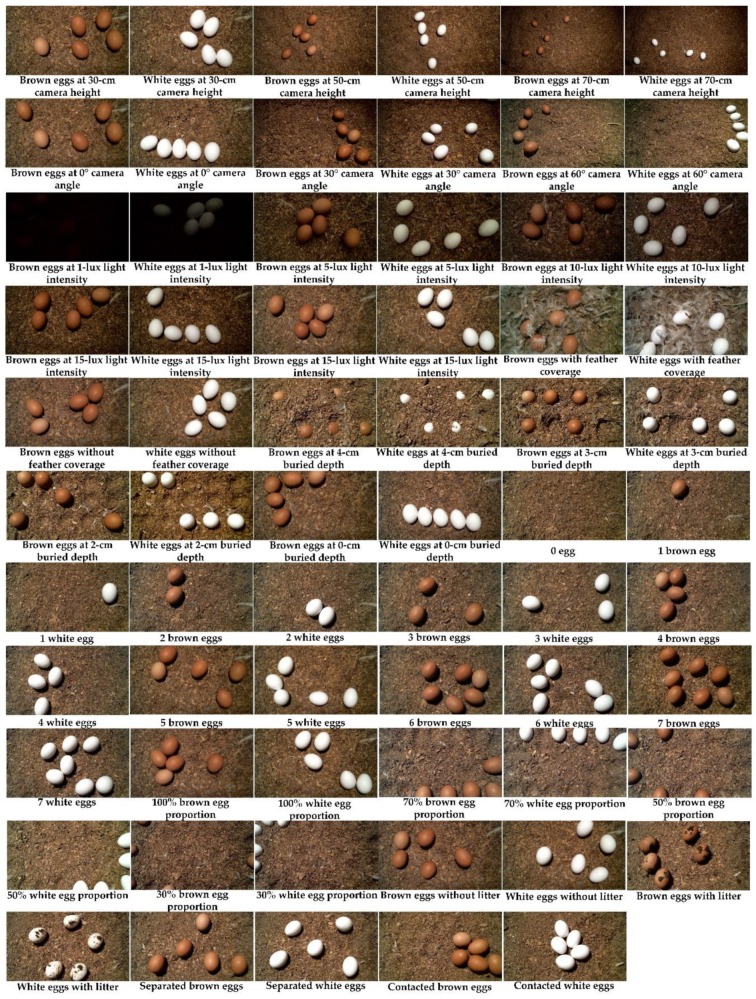
Sample images of floor eggs under various settings.

Table 1 and Figure 4 show the settings for the detector development, including camera height, camera tilting angle, light intensity, litter condition, egg color, buried depth, egg number in an image, egg proportion in an image, eggshell cleanness, and egg contact in an image. Each setting was examined with brown and white eggs. One hundred images were taken for each type of eggs in each level of settings. A total of 6,600 images (100 images per level of 33 settings for each type of eggs) were taken. As mentioned in Section 2.3, four-fifths of the images taken (5280 images) were used to train the three detectors for each of the five events. The configuration for the training is provided in Table 2. Each detector was trained with 200,000 iterations, beyond which the training loss was stable, as reported in TensorBoard, a TensorFlow visualization toolbox. The developed floor-egg detectors were saved as inference graphs and output as .pb files for further evaluation/testing.

### 2.5. Validation

#### 2.5.1. Validation Strategy 

As mentioned in Section 2.3, one-fifth of the images (1320 images) were used to validate the detectors for each of the five events. Average performance (precision, recall, accuracy, RMSE, and processing speed) for the three detector comparison and each level of settings via the optimal detector was calculated based on the five folds of the validation set. 

#### 2.5.2. Evaluation and Performance Metrics

**Figure 5:**
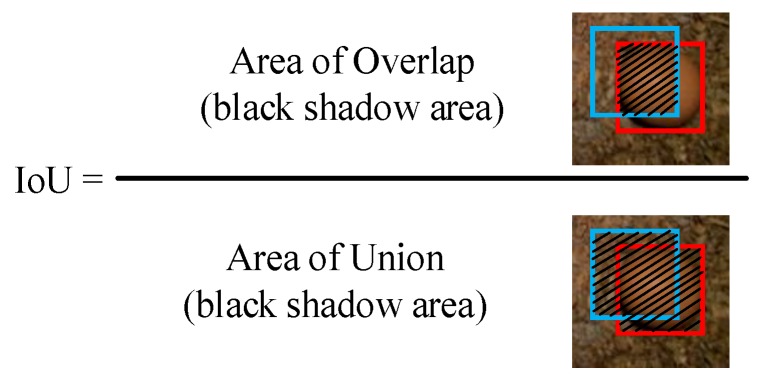
Illustration of intersection over union (IoU) calculation. The red square box is the ground truth box, the blue square box is the predicted box, and the black shadow areas are the overlap or union areas.

To determine whether an egg had been correctly detected, the intersection over union (IoU) for each bounding box was computed using overlap and union areas of the ground truth box and predicted box. Calculation of the IoU is illustrated in Figure 5. An IoU greater than 0.5 means the detector reported an egg correctly. 

Precision, recall, and accuracy for detecting each egg in the images were calculated using Equations (1)–(3). Precision is the ratio of correctly-predicted positives to the total predicted positives [23]. A higher precision suggests that a detector is less likely to identify a non-egg object as an egg. Recall refers the ratio of correctly-predicted positives to the total number of manually-labelled objects [23]. A higher recall suggests that a detector is less likely to miss floor egg detection. Accuracy is the ratio of correctly-predicted positives and negatives to the total detections [24]. A higher accuracy reflects better overall performance in detecting floor eggs and excluding non-egg objects.

(1) $$PRC=\frac{TP}{TP+FP}$$

(2) $$RCL=\;\frac{TP}{TP+FN}$$

(3) $$\mathrm{ACC}=\;\frac{TP+TN}{TP+FP+FN+TN}$$

where *PRC* is precision; *RCL* is recall; *ACC* is accuracy; *TP* is true positive, i.e., number of cases that a detector successfully detects an existent egg in an image with IoU greater than 0.5; *FP* is false positive, i.e., number of cases that a detector reports a nonexistent egg in an image, or IoU is less than 0.5; *FN* is false negative, i.e., number of cases that a detector fails to detect an existent egg in an image; and *TN* is true negative, i.e., number of cases that no egg is reported by both detector and manual label. 

Root mean square error (RMSE) of the egg location predicted by the detectors was calculated using Equations (4)–(6). The RMSE reflects the location deviation of a predicted egg from its actual location [25].

(4) $$RMSE_{x}=\;\sqrt{\frac{\sum_{i=1}^{N}{\left({\hat{x}}_{i}-x_{i}\right)}^{2}}{N}}$$

(5) $$RMSE_{y}=\;\sqrt{\frac{\sum_{i=1}^{N}{\left({\hat{y}}_{i}-y_{i}\right)}^{2}}{N}}$$

(6) $$RMSE_{xy}=\;\sqrt{\frac{\sum_{i=1}^{N}\left\{{\left({\hat{x}}_{i}-x_{i}\right)}^{2}+{\left({\hat{y}}_{i}-y_{i}\right)}^{2}\right\}}{N}}$$

where *RMSE_x_*, *RMSE_y_*, and *RMSE_xy_* are root mean square errors of the predicted egg center in horizontal (*x*), vertical (*y*), and actual directions, respectively; ${\hat{x}}_{i}$ and ${\hat{y}}_{i}$ are predicted center coordinates of the *i*th egg; $x_{i}$ and $y_{i}$ are the *i*th manually-labelled center coordinates; and *N* is the total number of eggs in the images. 

The processing time reported by Python 3.6 was used to evaluate the processing speed of the three CNN floor-egg detectors for processing 1320 images. The processing speed (ms·image^−1^) was obtained by dividing the total processing time with 1320 images. 

### 2.6. Comparison of Convolutional Neural Network (CNN) Floor-Egg Detectors

Precision, recall, accuracy, RMSE, and processing speed of floor egg detection via the three detectors were calculated within each of the five events using validation dataset. The performance was then averaged and compared to determine the optimal one. The settings for the three detector comparison were mentioned in Section 2.4.2, and the calculation procedures of the performance were described in Section 2.5.2. One-fifth of the images (1320 images) in each event were used for the evaluation. The pixel-to-distance conversion factors for calculating RMSEs were estimated as 0.19 mm/pixel for 30-cm camera height, 0.31 mm/pixel for 50-cm camera height, and 0.49 mm/pixel for 70-cm camera height. 

### 2.7. Evaluation of the Optimal Floor-Egg Detector under Different Settings

Based on the comparison of processing speed and accuracy for the three CNN floor-egg detectors, the optimal one was selected. The performance of the optimal one in floor egg detection was further evaluated under different settings, as mentioned in Section 2.4.2. The validation set, i.e., as mentioned in Section 2.3, one-fifth of the images (1320 images) in each event, were used for the evaluation. For each level of settings, average precision, recall, accuracy, *RMSE_x_*, *RMSE_y_*, and *RMSE_xy_* were calculated. 

### 2.8. Generalizability of the Optimal CNN Floor-Egg Detector

The performance of an object detector developed and evaluated under the same set of environments may not be informative in seeking to understand its performance under a new environment. To evaluate the detector generalizability, the performance of the optimal CNN detector was evaluated under new and random settings, i.e., the testing set mentioned in Section 2.3. The camera was installed at 25 cm above the litter with its lens pointing downward to capture top views of white or brown eggs. The light intensity was randomly set to 5–20 lux at bird level. Three to nine eggs buried at 0–4 cm depth in litter were captured in the images. The litter was mixed with feathers. Eggs were either contaminated with or without litter and contacted or separated in the images. Different proportions (30–100%) of eggs were randomly presented in an image. Three hundred (300) pictures were taken and used for evaluating the optimal floor-egg detector using the same metrics (Precision, recall, accuracy, *RMSE_x_*, *RMSE_y_*, and *RMSE_xy_*) described in Section 2.5.2. The pixel-to-distance conversion factor for calculating RMSE was 0.15 mm/pixel for a 25-cm camera height.

## 3. Results

### 3.1. Floor Egg Detection Using the CNN Floor-Egg Detectors

**Figure 6:**
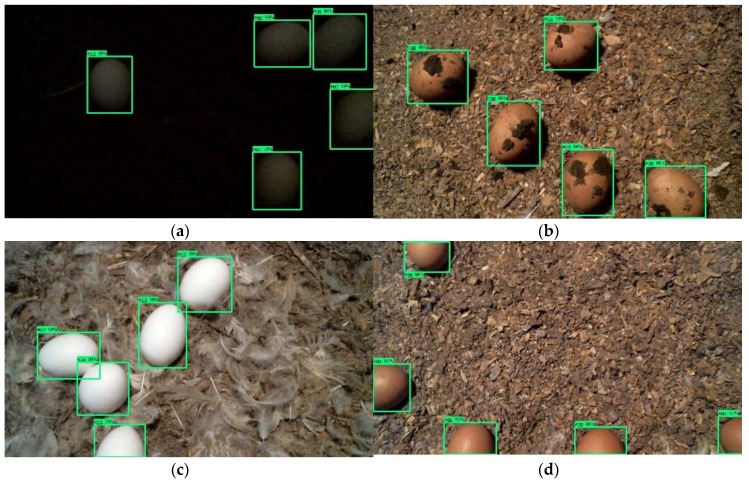
Sample images of floor egg detection under different settings: (**a**) five white eggs at the 1-lux light intensity; (**b**) five brown eggs with litter contamination; (**c**) five white eggs on the feather-mixed litter; (**d**) 50% of brown egg proportion in an image. Eggs in the images are detected and enclosed with green bounding boxes.

Figure 6 shows some sample images of floor egg detection using the newly-developed CNN floor-egg detectors. The setting of the sample images included five white eggs at the 1-lux light intensity, five brown eggs with litter contamination, five white eggs on the feather-mixed litter, and 50% of brown egg proportion in an image. Eggs in the images were identified by the CNN floor-egg detectors and enclosed in green bounding boxes. The coordinates of the bounding boxes can be readily extracted and used to locate eggs in the images, which provides inputs to control robots for floor egg collection.

### 3.2. Performance of the Three CNN Floor-Egg Detectors

Table 3 shows the performance of the SSD, faster R-CNN, and R-FCN detectors using five folds of the validation sets. Among the three detectors, the SSD detector had the fastest processing speed (125.1 ± 2.7 ms·image^−1^) and highest precision (99.9 ± 0.1%) but the lowest recall (72.1 ± 7.2%) and accuracy (72.0 ± 7.2%) and the highest RMSEs (1.0–1.4 mm). The R-FCN detector had the highest recall (98.5 ± 0.5%) but the lowest precision (93.3 ± 2.4%) and slowest processing speed (243.2 ± 1.0 ms·image^−1^). The faster R-CNN detector had the highest recall (98.4 ± 0.4%) and accuracy (98.1 ± 0.3%), low RMSEs (0.8–1.1 mm), and a medium processing speed (201.5 ± 2.3 ms·image^−1^). Because the faster R-CNN detector had great precision, recall, accuracy, RMSEs, and decent processing speed, it was selected as the optimal floor-egg detector.

### 3.3. Performance of the Optimal Convolutional Neural Network (CNN) Floor-Egg Detector

#### 3.3.1. Detector Performance with Different Camera Settings

Table 4 shows the precision, recall, accuracy, and RMSEs of floor egg detection by the optimal CNN, or faster R-CNN, floor-egg detector based on the five folds of the validation sets. The averaged precision, recall, and accuracy were above 97.6% for all camera settings in terms of both brown and white egg detection. The averaged RMSEs were 0.6–8.9 mm for brown and white egg detection and similar in horizontal (*x*) and vertical (*y*) directions. For brown and white egg detection, the RMSE results show that the deviation of the predicted egg center from the actual egg center increased at larger camera heights and tilting angles. 

#### 3.3.2. Detector Performance with Different Environmental Settings

Table 5 shows the precision, recall, accuracy, and RMSEs of the faster R-CNN detector for floor egg detection under different light intensities (1, 5, 10, 15, and 20 lux) and litter conditions (w/or w/o feather presence) using the five folds of the validation sets. The detector generally performed greatly at most light intensities; however, the recall and accuracy of the detector for brown egg detection were poor (less than 35%) at the 1-lux light intensity. The RMSEs were 0.5–1.5 mm for most of the environmental settings. The highest RMSE values (2.3, 2.9, and 4.5 mm in horizontal (*x*), vertical (*y*), and actual directions, respectively) were observed at the 1-lux light intensity for brown egg detection.

#### 3.3.3. Detector Performance with Different Egg Settings

Table 6 shows the precision, recall, accuracy, and RMSEs of brown and white egg detection by the faster R-CNN detector under different egg settings using the five folds of the validation sets. Precision, recall, and accuracy were over 97% for all of the egg settings. The largest RMSEs (2.1 mm) were present at the largest buried depth (4 cm) and the smallest egg proportion in an image (30%) for brown eggs. The RMSEs were 0.4–1.9 mm and were not affected by most egg settings.

### 3.4. Performance of the Faster R-CNN Detector under Random Settings

Table 7 shows the precision, recall, accuracy, and RMSEs of brown and white egg detection by the faster R-CNN detector under random settings (Section 2.8). Precision, recall, and accuracy were above 94.5% for brown egg detection, however, precision and accuracy decreased to 91.9% for white egg detection. The RMSEs were 1.0–1.4 mm for brown eggs and 0.9–1.3 mm for white eggs.

## 4. Discussion

Three CNN floor-egg detectors were developed in this study and evaluated for their performance on floor egg detection based on a five-fold cross-validation strategy. The settings used for training and validation consisted of a wide range of common CF housing conditions under which the performance of the optimal floor-egg detector was further evaluated. Our results show that the optimal detector performed almost perfectly (Table 4, Table 5 and Table 6) for egg detection when it was trained and validated using the dataset obtained from the same CF conditions. Another set of images taken under randomly-selected CF housing conditions was used to test the generalizability of the optimal floor-egg detector. It should be noted that performance of the floor-egg detectors was evaluated under simulated CF environments in this study, and thus requires further validation under real commercial CF environments. 

### 4.1. Performance of the Three CNN Floor-Egg Detectors

**Figure 7:**
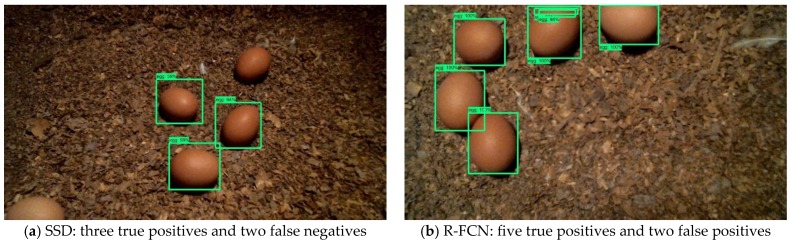
Sample images of erroneous floor egg detection by the SSD (**a**) and R-FCN (**b**) detectors. Eggs in the images are detected and enclosed with green bounding boxes.

In this study, a comparison of the three CNN floor-egg detectors showed that the faster R-CNN detector had the highest precision, recall, and accuracy among the three detectors to detect floor eggs under a wide range of CF housing conditions. The precision of the SSD detector and recall of the R-FCN detector were comparable to those of the faster R-CNN detector; however, the recall of the SSD detector, precision of the R-FCN detector, and accuracy of these two detectors were subpar, especially for the SSD. Based on our observations, the main reason of subpar performance of the SSD and R-FCN detectors were the underprediction (by the SSD detector, Figure 7a) and overprediction (by the R-FCN detector, Figure 7b) of egg numbers in the images. Huang, et al. [11], Zhang, et al. [26], and Pacha, et al. [27] also reported that the SSD and R-FCN performed less accurately than faster R-CNN in terms of detecting other types of objects (e.g., handwritten music symbol, wild animal, etc.). Because the SSD detector (with Mobilenet V1 feature extractor) had fewer layers than the other two CNN detectors, it could not obtain as many floor egg features as its counterparts [28]. This resulted in the miss-identification of floor eggs in the images. The R-FCN detector with the Resnet101 feature extractor had more layers than the other two detectors but could be oversensitive to feature extraction [13]. As such, the R-FCN detector was more likely to misidentify non-egg objects as eggs than the other two detectors. Overall, the faster R-CNN detector performed the best in terms of precision, recall, and accuracy among the three detectors. 

The faster R-CNN and R-FCN detectors were more accurate for locating the floor eggs in the images, as indicated by their smaller RMSEs, compared to the SSD detector. Considering a regular egg size to be 55 mm in length and 42 mm in width [29], the RMSEs in egg detection can be translated into egg center detection deviations of 1–3% for the faster R-CNN and R-FCN detectors. 

When selecting a CNN floor-egg detector for an egg collection robot, the processing speed of the detector should be considered, because it determines the robot operations, such as running speed. Among the three CNN detectors, the SSD detector had the fastest processing speed, while the R-FCN detector had the slowest one. Huang, et al. [11] found that the SSD detector had the fastest processing speed among these three detectors. Okafor, et al. [30] reported that SSD processed images more quickly than faster R-CNN (662.3–900.9 ms·image^−1^ vs. 1162.8–1470.6 ms·image^−1^). Network complexity may influence the processing speed. The SSD detector had a one-stage convolutional network and the most lightweight feature extractor (Mobilenet V1) of the three extractors [12,19]; therefore, it processed the images most quickly among the three detectors. In contrast, the R-FCN detector had two-stage convolutional network and the deepest feature extractor (Resnet101) [13,21], which compromised the processing speed. Although the processing speed of the faster R-CNN detector (201.5 ms·image^−1^ or 5 images·s^−1^) was slower than that of the SSD detector, it can be improved by decreasing input image sizes and number of region proposals [11] and by upgrading the PC hardware [31]. 

### 4.2. Performance of the Faster R-CNN Detector under Different Settings

Precision, recall, and accuracy of the optimal floor-egg detector, or the faster R-CNN detector, were determined under a wide range of CF housing conditions (Section 2.4.2). The three parameters were over 97% for most of the conditions. The recall and accuracy of the faster R-CNN detector for brown egg detection at the 1-lux light intensity were low but could be improved by integrating lamps to light up dark areas when incorporating the detector to egg-collecting robots. The RMSEs of the faster R-CNN detector increased profoundly with camera height, reflecting larger deviations of predicted egg centers from actual egg centers when the camera was placed higher above the floor eggs. The reason for this is that the pixel-to-distance conversion ratio (mm/pixel) increases at a higher camera placement, which results in a larger distance deviation for the same pixel prediction error by the detector [32]. The pixel-to-distance conversion ratios at the 50-cm and 70-cm camera heights are, respectively, 1.6 and 2.6 times more than that at the 30-cm camera height. 

### 4.3. Performance of the Faster R-CNN Detector under Random Settings

As expected, some performance metrics of the faster R-CNN detector decreased when the detector was evaluated under random settings (Section 2.8). Specifically, precision decreased from 97.3–99.9% (the detector was trained and validated using the dataset from the same CF conditions; see Section 2.4.2) to 91.9–94.7% for brown and white egg detection under random settings, and accuracy decreased from 99.2–99.9% to 91.9–94.5%. Based on our observation, the drop of precision was due to more misidentifications of feather to eggs. Such misidentifications also compromised the accuracy of the detector. Recall remained high (over 99%) for both types of eggs, which meant that the detector was less likely to miss-identify existent eggs in an image under random settings. Vroegindeweij, et al. [4] developed a vision system based on image processing for an egg-collecting robot. Their image processing algorithms were parameterized according to specific egg shape, color, and size. The system achieved 86% accuracy in the detection of floor eggs under a laboratory CF housing; however, the generalizability of the system (i.e., the accuracy when using the system under different CF housing conditions) was not described. The faster R-CNN detector developed in this study can easily be generalized to random conditions, making it a powerful tool with which to handle variable CF environments.

## 5. Conclusions

Three CNN floor-egg detectors (single shot detector “SSD”, faster region-based convolutional neural network “faster R-CNN”, and region-based fully convolutional network “R-FCN”) were developed and compared in terms of processing time, precision, recall, accuracy, and root mean square error (RMSE). The optimal floor-egg detector was selected for further performance evaluation on floor egg detection under various cage-free (CF) housing conditions. The following conclusions can be drawn based on the results of this study:

- Compared with the SSD and R-FCN detectors, the faster R-CNN detector had better recall (98.4 ± 0.4%) and accuracy (98.1 ± 0.3%) for detecting floor eggs under a wide range of commercial conditions and system setups. It also had decent processing speed (201.5 ± 2.3 ms·image^−1^), precision (99.7 ± 0.2%), and RMSE (0.8–1.1 mm) for the detection.
- The faster R-CNN detector performed very well in detecting floor eggs under a range of common CF housing conditions, except for brown eggs at the 1-lux light intensity. Its performance was not affected by camera height, camera tilting angle, light intensity, litter condition, egg color, buried depth, egg number in an image, egg proportion in an image, eggshell cleanness, or egg contact in images.
- The precision, recall, and accuracy of the faster R-CNN detector in floor egg detection were 91.9%–100% under random settings, suggesting good generalizability.

The developed faster R-CNN floor egg detection will be integrated into an egg-collecting robot for automated floor detection and collection in the CF hen housing systems. Lamps to light up dark area and blowers to blow feather atop floor eggs in the CF housing system will be installed onto the robot to improve floor egg detection performance. Floor egg detection speed via the faster R-CNN floor egg detector will be further validated with robot operation frequency to determine the detector efficiency on floor egg detection.

## Figures and Tables

**Table 1:** Settings for evaluating the optimal convolutional neural network (CNN) floor-egg detector.

Setting		Level
Camera settings	Camera height	30, 50, and 70 cm
	Camera tilting angle	0, 30, and 60°
Environmental settings	Light intensity	1, 5, 10, 15, and 20 lux
	Litter condition	with and without feather
Egg settings	Buried depth	0, 2, 3, and 4 cm
	Egg number in an image	0, 1, 2, 3, 4, 5, 6, and 7
	Egg proportion in an image	30%, 50%, 70%, and 100%
	Eggshell cleanness	with and without litter
	Egg contact in an image	contacted and separated

Note: Camera tilting angle is the dihedral angle between the plane of the camera and the horizontal plane. All tests were conducted under the following conditions: 30-cm camera height, top camera view, 20-lux light intensity, litter without feather, and five eggs (white or brown), unless specified in ‘Level’.

**Table 2:** Configuration for training the Convolutional Neural Network (CNN) floor-egg detectors.

Parameters	CNN Floor-Egg Detectors		
	SSD	Faster R-CNN	R-FCN
Batch size	24	1	1
Initial learning rate	4.0 × 10^−3^	2.0 × 10^−4^	3.0 × 10^−4^
Learning rate at 90,000 steps	3.6 × 10^−3^	2.0 × 10^−5^	3.0 × 10^−5^
Learning rate at 120,000 steps	3.2 × 10^−3^	2.0 × 10^−6^	2.0 × 10^−6^
Momentum optimizer value	0.9	0.9	0.9
Epsilon value	1.0	–	–
Gradient clipping by norm	–	10.0	10.0

Note: SSD is single shot detector, faster R-CNN is faster region-based convolutional neural network, and R-FCN is region-based fully convolutional network.

**Table 3:** Performance (mean ± standard deviation) of three convolutional neural network (CNN) floor-egg detectors on floor egg detection using 1320 images in each of the five events.

Detector	Processing Speed (ms·image^−1^)	PRC (%)	RCL (%)	ACC (%)	RMSE (mm)		
					*RMSE_x_*	*RMSE_y_*	*RMSE_xy_*
SSD	125.1 ± 2.7	99.9 ± 0.1	72.1 ± 7.2	72.0 ± 7.2	1.0 ± 0.1	1.0 ± 0.1	1.4 ± 0.1
Faster R-CNN	201.5 ± 2.3	99.7 ± 0.2	98.4 ± 0.4	98.1 ± 0.3	0.8 ± 0.1	0.8 ± 0.1	1.1 ± 0.1
R-FCN	243.2 ± 1.0	93.3 ± 2.4	98.5 ± 0.5	92.0 ± 2.5	0.8 ± 0.1	0.8 ± 0.1	1.1 ± 0.1

Note: SSD is single shot detector; Faster R-CNN is faster region-based convolutional neural network; R-FCN is region-based fully convolutional network; PRC is precision; RCL is recall; ACC is accuracy; RMSE is root mean square error; and *RMSE_x_*, *RMSE_y_*, and *RMSE_xy_* are root mean square errors of predicted egg center in horizonal (*x*), vertical (*y*), and actual directions, respectively. The performance in the table was calculated based on the validation sets.

**Table 4:** Performance (mean ± standard deviation) of the faster region-based convolutional neural network (faster R-CNN) floor-egg detector on floor egg detection under different camera settings using average 40 images of each level of the settings in each of the five events.

Settings		Brown Egg						White Egg					
		PRC (%)	RCL (%)	ACC (%)	RMSE (mm)			PRC (%)	RCL (%)	ACC (%)	RMSE (mm)		
					*RMSE_x_*	*RMSE_y_*	*RMSE_xy_*				*RMSE_x_*	*RMSE_y_*	*RMSE_xy_*
Camera height (cm)	30	99.6 ± 0.7	99.9 ± 0.1	99.9 ± 0.1	0.8 ± 0.1	0.6 ± 0.1	1.2 ± 0.1	97.6 ± 2.7	99.9 ± 0.1	99.9 ± 0.1	0.8 ± 0.1	0.6 ± 0.1	1.0 ± 0.1
	50	99.8 ± 0.2	99.9 ± 0.1	99.9 ± 0.1	1.7 ± 0.3	1.4 ± 0.4	2.0 ± 0.6	99.6 ± 0.5	99.9 ± 0.1	99.9 ± 0.1	2.0 ± 0.6	1.5 ± 0.7	3.3 ± 0.9
	70	99.7 ± 0.4	99.9 ± 0.1	99.9 ± 0.1	4.9 ± 0.9	5.8 ± 1.1	8.0 ± 0.9	98.9 ± 1.0	99.9 ± 0.1	99.9 ± 0.1	6.5 ± 1.1	6.1 ± 0.9	8.9 ± 1.0
Camera tilting angle (°)	0	99.9 ± 0.1	99.9 ± 0.1	99.9 ± 0.1	0.7 ± 0.1	0.6 ± 0.1	0.9 ± 0.2	99.8 ± 0.4	99.9 ± 0.1	99.9 ± 0.1	0.7 ± 0.1	0.6 ± 0.1	1.0 ± 0.2
	30	99.7 ± 0.5	99.9 ± 0.1	99.9 ± 0.1	1.4 ± 0.1	0.8 ± 0.1	1.6 ± 0.2	99.1 ± 0.9	99.9 ± 0.1	99.9 ± 0.1	0.9 ± 0.1	0.7 ± 0.1	1.3 ± 0.2
	60	99.8 ± 0.4	99.7 ± 0.5	99.7 ± 0.5	1.9 ± 0.1	1.9 ± 0.1	2.5 ± 0.2	99.9 ± 0.1	99.9 ± 0.1	99.9 ± 0.1	1.5 ± 0.1	1.0 ± 0.1	1.8 ± 0.2

Note: Camera tilting angle is the dihedral angle between camera lens plane and horizontal plane; PRC is precision; RCL is recall; ACC is accuracy; and *RMSE_x_*, *RMSE_y_*, and *RMSE_xy_* are root mean square errors of predicted egg center in horizonal (*x*), vertical (*y*), and actual directions, respectively. The performance in the table was calculated based on the validation sets.

**Table 5:** Performance (mean ± standard deviation) of the faster region-based convolutional neural network (faster R-CNN) floor-egg detector on floor egg detection under different environmental settings using average 40 images of each level of the settings in each of the five events.

Settings		Brown Egg						White Egg					
		PRC (%)	RCL (%)	ACC (%)	RMSE (mm)			PRC (%)	RCL (%)	ACC (%)	RMSE (mm)		
					*RMSE_x_*	*RMSE_y_*	*RMSE_xy_*				*RMSE_x_*	*RMSE_y_*	*RMSE_xy_*
Light intensity (lux)	1	98.2 ± 1.8	34.4 ± 7.9	34.4 ± 7.9	2.3 ± 0.4	2.9 ± 0.4	4.5 ± 0.6	99.9 ± 0.1	99.9 ± 0.1	99.9 ± 0.1	0.8 ± 0.4	1.3 ± 0.3	1.5 ± 0.6
	5	99.9 ± 0.1	99.9 ± 0.1	99.9 ± 0.1	1.0 ± 0.1	1.0 ± 0.1	1.4 ± 0.2	99.6 ± 0.9	99.9 ± 0.1	99.9 ± 0.1	0.7 ± 0.1	1.0 ± 0.1	1.4 ± 0.2
	10	98.8 ± 1.6	99.9 ± 0.1	99.9 ± 0.1	0.7 ± 0.1	0.7 ± 0.1	1.1 ± 0.1	99.8 ± 0.3	99.9 ± 0.1	99.9 ± 0.1	0.8 ± 0.1	0.5 ± 0.1	0.9 ± 0.2
	15	99.8 ± 0.3	99.9 ± 0.1	99.9 ± 0.1	0.8 ± 0.1	0.7 ± 0.1	1.2 ± 0.1	99.9 ± 0.1	99.9 ± 0.1	99.9 ± 0.1	0.9 ± 0.1	0.7 ± 0.1	1.1 ± 0.1
	20	99.9 ± 0.1	99.9 ± 0.1	99.9 ± 0.1	0.9 ± 0.1	0.5 ± 0.1	1.1 ± 0.1	99.9 ± 0.1	99.9 ± 0.1	99.9 ± 0.1	0.8 ± 0.1	0.7 ± 0.1	1.1 ± 0.1
Litter condition	w/feather	99.0 ± 1.3	99.9 ± 0.1	99.9 ± 0.1	1.4 ± 0.3	1.2 ± 0.2	1.7 ± 0.3	99.6 ± 0.7	99.9 ± 0.1	99.9 ± 0.1	0.7 ± 0.1	0.8 ± 0.1	1.1 ± 0.1
	w/o feather	99.9 ± 0.1	99.9 ± 0.1	99.9 ± 0.1	0.9 ± 0.1	0.8 ± 0.1	1.1 ± 0.1	99.9 ± 0.1	99.9 ± 0.1	99.9 ± 0.1	0.5 ± 0.1	0.9 ± 0.1	1.0 ± 0.1

Note: PRC is precision; RCL is recall; ACC is accuracy; RMSE is root mean square error; and *RMSE_x_*, *RMSE_y_*, and *RMSE_xy_* are root mean square errors of predicted egg center in horizonal (*x*), vertical (*y*), and actual directions, respectively. The performance in the table was calculated based on the validation sets.

**Table 6:** Performance (mean ± standard deviation) of the faster region-based convolutional neural network (faster R-CNN) floor-egg detector on floor egg detection under different egg settings using an average of 40 images of each level of the settings in each of the five events.

Settings		Brown Egg						White Egg					
		PRC (%)	RCL (%)	ACC (%)	RMSE (mm)			PRC (%)	RCL (%)	ACC (%)	RMSE (mm)		
					*RMSE_x_*	*RMSE_y_*	*RMSE_xy_*				*RMSE_x_*	*RMSE_y_*	*RMSE_xy_*
Buried depth (cm)	0	99.9 ± 0.1	99.9 ± 0.1	99.9 ± 0.1	0.7 ± 0.1	0.7 ± 0.1	1.0 ± 0.1	99.9 ± 0.1	99.9 ± 0.1	99.9 ± 0.1	0.8 ± 0.1	0.7 ± 0.1	1.0 ± 0.1
	2	99.7 ± 0.7	99.7 ± 0.5	99.9 ± 0.1	0.6 ± 0.1	1.5 ± 0.1	1.8 ± 0.1	98.6 ± 1.5	99.6 ± 0.8	99.9 ± 0.1	0.7 ± 0.1	0.9 ± 0.1	1.1 ± 0.1
	3	99.9 ± 0.1	99.8 ± 0.2	99.9 ± 0.1	0.8 ± 0.1	0.8 ± 0.1	1.2 ± 0.1	99.3 ± 0.6	99.6 ± 0.7	99.9 ± 0.1	0.9 ± 0.1	0.6 ± 0.1	1.0 ± 0.1
	4	99.9 ± 0.1	99.2 ± 1.1	99.6 ± 0.8	1.6 ± 0.2	1.3 ± 0.2	2.1 ± 0.2	99.6 ± 0.9	99.8 ± 0.4	99.9 ± 0.1	0.9 ± 0.1	1.6 ± 0.1	1.9 ± 0.2
Egg number in an image	0	–	–	99.9 ± 0.1	–	–	–	–	–	99.9 ± 0.1	–	–	–
	1	98.0 ± 4.4	99.9 ± 0.1	99.9 ± 0.1	0.7 ± 0.3	0.9 ± 0.3	1.1 ± 0.4	97.3 ± 3.6	99.9 ± 0.1	99.9 ± 0.1	1.0 ± 0.2	0.9 ± 0.2	1.1 ± 0.3
	2	99.1 ± 1.9	99.9 ± 0.1	99.9 ± 0.1	0.9 ± 0.2	0.6 ± 0.1	1.1 ± 0.2	99.9 ± 0.1	99.9 ± 0.1	99.9 ± 0.1	0.8 ± 0.1	0.6 ± 0.1	1.0 ± 0.1
	3	99.9 ± 0.1	99.9 ± 0.1	99.9 ± 0.1	0.8 ± 0.1	0.8 ± 0.1	1.1 ± 0.1	99.9 ± 0.1	99.9 ± 0.1	99.9 ± 0.1	0.9 ± 0.2	0.6 ± 0.1	1.1 ± 0.1
	4	99.9 ± 0.1	99.9 ± 0.1	99.9 ± 0.1	0.7 ± 0.1	0.7 ± 0.1	1.0 ± 0.1	99.9 ± 0.1	99.9 ± 0.1	99.9 ± 0.1	0.8 ± 0.1	0.6 ± 0.1	1.0 ± 0.1
	5	99.9 ± 0.1	99.9 ± 0.1	99.9 ± 0.1	0.8 ± 0.1	0.7 ± 0.1	1.0 ± 0.1	99.9 ± 0.1	99.9 ± 0.1	99.9 ± 0.1	0.7 ± 0.1	0.4 ± 0.1	0.9 ± 0.1
	6	99.9 ± 0.1	99.9 ± 0.1	99.9 ± 0.1	0.8 ± 0.1	0.8 ± 0.1	1.1 ± 0.1	99.9 ± 0.1	99.9 ± 0.1	99.9 ± 0.1	0.5 ± 0.1	0.6 ± 0.1	0.8 ± 0.1
	7	99.9 ± 0.1	99.9 ± 0.1	99.9 ± 0.1	1.5 ± 0.1	0.9 ± 0.1	1.7 ± 0.2	99.9 ± 0.1	99.9 ± 0.1	99.9 ± 0.1	0.9 ± 0.1	0.6 ± 0.1	1.0 ± 0.1
Egg proportion in an image (%)	30	99.8 ± 0.5	99.3 ± 0.9	99.3 ± 0.9	1.7 ± 0.3	1.2 ± 0.1	2.1 ± 0.3	99.6 ± 0.3	99.5 ± 0.4	99.6 ± 0.6	0.7 ± 0.1	0.6 ± 0.1	1.0 ± 0.1
	50	99.9 ± 0.1	99.9 ± 0.1	99.9 ± 0.1	1.0 ± 0.2	0.9 ± 0.1	1.3 ± 0.2	99.9 ± 0.1	99.9 ± 0.1	99.9 ± 0.1	0.8 ± 0.1	0.6 ± 0.1	1.0 ± 0.1
	70	99.9 ± 0.1	99.3 ± 0.9	99.9 ± 0.1	0.8 ± 0.3	0.8 ± 0.1	1.0 ± 0.1	99.9 ± 0.1	99.6 ± 0.5	99.9 ± 0.1	0.7 ± 0.1	0.7 ± 0.1	0.9 ± 0.1
	100	99.9 ± 0.1	99.9 ± 0.1	99.9 ± 0.1	0.5 ± 0.1	0.6 ± 0.1	0.8 ± 0.2	99.9 ± 0.1	99.9 ± 0.1	99.9 ± 0.1	0.7 ± 0.1	0.7 ± 0.1	1.0 ± 0.1
Eggshell cleanness	w/litter	99.9 ± 0.1	99.5 ± 0.8	99.9 ± 0.1	0.9 ± 0.1	1.2 ± 0.2	1.6 ± 0.2	99.9 ± 0.1	99.8 ± 0.4	99.9 ± 0.1	0.7 ± 0.1	0.7 ± 0.1	1.0 ± 0.1
	w/o litter	99.9 ± 0.1	99.9 ± 0.1	99.9 ± 0.1	0.7 ± 0.1	0.7 ± 0.1	1.0 ± 0.1	99.9 ± 0.1	99.9 ± 0.1	99.9 ± 0.1	0.8 ± 0.1	0.7 ± 0.1	1.1 ± 0.1
Egg contact in an image	contacted	99.9 ± 0.1	99.9 ± 0.2	99.9 ± 0.1	0.8 ± 0.2	0.8 ± 0.1	1.2 ± 0.1	99.6 ± 0.8	99.2 ± 1.6	99.2 ± 1.8	0.9 ± 0.1	0.7 ± 0.1	1.1 ± 0.1
	separated	99.9 ± 0.1	99.8 ± 0.4	99.9 ± 0.1	0.8 ± 0.1	1.4 ± 0.1	1.6 ± 0.2	99.9 ± 0.1	99.6 ± 0.8	99.9 ± 0.1	0.7 ± 0.1	0.8 ± 0.1	0.9 ± 0.1

Note: PRC is precision; RCL is recall; ACC is accuracy; RMSE is root mean square error; RMSE is root mean square error; and *RMSE_x_*, *RMSE_y_*, and *RMSE_xy_* are root mean square errors of predicted egg center in horizonal (*x*), vertical (*y*), and actual directions, respectively. The performance in the table was calculated based on the validation sets.

**Table 7:** Performance of the faster region-based convolutional neural network (faster R-CNN) floor-egg on floor egg detection under random settings using 150 images for each type of eggs.

Brown Egg						White Egg					
PRC (%)	RCL (%)	ACC (%)	RMSE (mm)			PRC (%)	RCL (%)	ACC (%)	RMSE (mm)		
			*RMSE_x_*	*RMSE_y_*	*RMSE_xy_*				*RMSE_x_*	*RMSE_y_*	*RMSE_xy_*
94.7	99.8	94.5	1.0	1.1	1.4	91.9	100.0	91.9	0.9	0.9	1.3

Note: PRC is precision; RCL is recall; ACC is accuracy; RMSE is root mean square error; and *RMSE_x_*, *RMSE_y_*, and *RMSE_xy_* are root mean square errors of predicted egg center in horizonal (*x*), vertical (*y*), and actual directions, respectively. The performance in the table was calculated based on the testing set.
